# Sarcopenia in Informal Caregivers of Persons Living with Dementia: A Scoping Review

**DOI:** 10.3390/jcm15197345

**Published:** 2026-09-22

**Authors:** Kai Heng Raymond Goh, Qin Kimberly Tjahjadi Tsai, Xiaoting Huang

**Affiliations:** 1 Department of Post Acute and Continuing Care, Jurong Community Hospital, Singapore 609606, Singapore; 2 Department of Internal Medicine, Singapore General Hospital, Singapore 169608, Singapore; 3 Department of Geriatric Medicine, Singapore General Hospital, Singapore 169608, Singapore

**Keywords:** sarcopenia, dementia caregiving, frailty, muscle strength, physical performance

## Abstract

**Background/Objectives**: Dementia caregiving is associated with psychological stress, but the impact on caregivers’ physical health, particularly sarcopenia—the progressive loss of muscle mass, strength, and function—remains under-explored. Sarcopenia is clinically significant as a predictor of falls and disability and is potentially modifiable through exercise and nutrition. This scoping review aimed to map existing evidence on sarcopenia and its components in informal dementia caregivers. **Methods**: This review followed Joanna Briggs Institute methodology and the PRISMA-ScR checklist. Five databases (PubMed/MEDLINE, Scopus, PsycINFO, ProQuest Dissertations & Theses, medRxiv) were searched from 2000 to July 2025 for studies of adult informal caregivers of persons with dementia reporting at least one sarcopenia domain (muscle mass, muscle strength, or physical performance). Screening and data charting were performed independently by two reviewers. No formal critical appraisal or meta-analysis was undertaken because the review aimed to map the extent, characteristics and methodological gaps of the available evidence. **Results**: Six sources were included: four cross-sectional studies, one retrospective cohort and one prospective cohort. No study used consensus definitions (EWGSOP2/AWGS); most examined surrogate markers such as handgrip strength or gait speed. Findings were heterogeneous: some studies suggested impaired physical function, frailty, and endurance deficits among caregivers, while others reported mixed results. Nutritional risk was evident in one study, with 23% malnourished and 41% at risk. No interventional studies were identified. **Conclusions**: The evidence base on sarcopenia in dementia caregivers is sparse and inconsistent, relying largely on proxy measures and cross-sectional data. Preliminary findings suggest that informal caregivers, especially older spousal caregivers, may be vulnerable to declines in muscle health and function. Future research should adopt standardised sarcopenia criteria, employ longitudinal designs, and test interventions such as resistance exercise and nutritional supplementation to mitigate risk.

## 1. Introduction

Dementia currently affects more than 55 million people worldwide [1], a number projected to double every 20 years. This trend ensures a continued expansion of the unpaid caregiving workforce. The psychological consequences of caregiving—depression, anxiety and caregiver burden—are well established [2]; yet the physical sequelae, particularly sarcopenia, have received comparatively little attention. Sarcopenia is defined as a generalised skeletal muscle disorder characterised by loss of muscle mass, strength and performance [3], which predicts falls, disability and premature mortality. Importantly, its pathogenesis is age-related and modifiable, making it a tractable target for prevention and intervention.

Focusing on sarcopenia in caregivers is conceptually and clinically distinct from the broader construct of frailty. Whereas frailty indices encompass multiple domains (weakness, weight loss, exhaustion, etc.) [4], sarcopenia isolates the musculoskeletal component, is diagnosable with objective consensus-based cut-points, and is directly responsive to interventions like resistance exercise and protein supplementation [3]. Frailty in dementia caregivers has been reported in cross-sectional studies [5], but those investigations rarely employed muscle-specific outcomes or stratified findings by dementia severity. By addressing sarcopenia specifically, we aim to identify an earlier, potentially reversible stage of physical decline, refine risk stratification, and facilitate mechanism-based interventions.

Two considerations underscore the plausibility of caregiver sarcopenia. First, caregiving tasks intensify as dementia progresses—from supervision in mild disease to near-constant physical assistance in severe disease—exposing caregivers to repetitive lifting, transfers, and other strains, often with disrupted self-care (e.g., missing exercise or medical appointments) [6]. Second, chronic caregiving stress activates neuroendocrine pathways (e.g., HPA-axis hypercortisolism) and pro-inflammatory cytokines that can accelerate muscle catabolism [7]. Sustained hypercortisolism has been shown to suppress anabolic signalling and promote skeletal muscle proteolysis, while chronically elevated interleukin-6 and tumour necrosis factor-alpha impair muscle protein synthesis. These mechanisms are well-documented in ageing and chronic stress physiology [7]. The convergence of mechanical overload and stress-related endocrine–inflammatory dysregulation therefore makes sarcopenia a plausible, if as yet unconfirmed, outcome of prolonged caregiving.

Caregivers—often older spouses—experience unique combinations of physical effort, sleep fragmentation, suboptimal diet and chronic stress that can erode muscle function [8]. Neuropsychiatric symptoms in dementia (agitation, wandering) further escalate nighttime activity and vigilance demands, amplifying caregivers’ physiological strain. Notably, women (who form the majority of primary caregivers) may be at heightened vulnerability due to age-associated hormonal changes and fewer opportunities for exercise. Despite these concerns, only a handful of studies to date have quantified muscle health outcomes in dementia caregivers [9,10,11,12,13,14]. Some cross-sectional analyses have documented higher frailty scores and lower grip strength in caregivers compared to non-caregiving controls [5,11,12,13,14]. However, most did not use formal sarcopenia criteria (e.g., European Working Group on Sarcopenia in Older People, EWGSOP) and lacked longitudinal follow-up [9,10,11,14]. A systematic appraisal of the literature is needed to (i) consolidate these disparate findings, (ii) expose methodological deficiencies (such as inconsistent muscle measures or cut-offs), and (iii) identify priorities for future research and interventions. This urgency is underscored in fast-ageing societies (e.g., Singapore) where nearly half of primary dementia caregivers are ≥60 years old, yet population-based data on their muscle health are absent.

The present scoping review was undertaken to map the breadth of evidence on sarcopenia and its key components (muscle mass, strength, and physical performance) in informal caregivers of persons living with dementia. We sought to clarify conceptual and methodological shortcomings in this literature and to guide future preventive and therapeutic research targeting caregiver sarcopenia. A scoping review approach was selected because the literature was expected to be sparse and methodologically heterogeneous, with variation in study designs, caregiver populations and measures of muscle health, making evidence mapping more appropriate than pooled effect estimation.

## 2. Materials and Methods

This scoping review followed the Joanna Briggs Institute (JBI) methodological guidance [15] for scoping reviews and is reported in accordance with the PRISMA-ScR (Preferred Reporting Items for Systematic reviews and Meta-Analyses extension for Scoping Reviews) [16] checklist. The completed PRISMA checklist can be found in Supplementary File S1. A protocol for this review was not prospectively registered or published.

We included English-language original empirical studies using quantitative, qualitative or mixed-methods designs that examined adult informal or unpaid caregivers of persons living with dementia and reported at least one cardinal sarcopenia-related domain: skeletal muscle mass, muscle strength or objective physical performance. We included studies of any design (e.g., cross-sectional, cohort, or intervention trials) given the expected paucity of data. We excluded publications focusing exclusively on professional caregivers or non-dementia caregiving contexts, conference abstracts without full text, and narrative/commentary articles without primary data. Operational definitions of sarcopenia only gained traction after 2010; however, we opted to search from 2000 onwards to capture any earlier relevant work that might not explicitly use the term “sarcopenia” but measured muscle outcomes.

A search strategy combining controlled vocabulary and free-text terms relating to informal caregivers, dementia and sarcopenia or its component domains was developed for PubMed and reviewed by a second reviewer against the PRESS checklist. PubMed, Scopus and APA PsycINFO were searched on 28 June 2025; ProQuest Dissertations & Theses Global was searched on 14 July 2025; and medRxiv was searched on 20 July 2025. Searches covered records published from 1 January 2000 to the date of the respective search. Syntax was adapted to the indexing and functionality of each information source. The complete PubMed strategy, including the limits applied, is provided in Supplementary Material S1. Reference lists of included sources were also examined for additional potentially eligible reports.

All retrieved citations were imported into Rayyan (Qatar Computing Research Institute) for de-duplication and screening. After automated and manual duplicate removal, two reviewers (GKHR and TQK) independently screened titles/abstracts for relevance. At this stage, any citation deemed potentially relevant by either reviewer advanced to full-text review. Full-text articles were then obtained and assessed against inclusion criteria by the same two reviewers, working independently and in a blinded fashion. Disagreements were resolved through discussion, with a third reviewer (XH) available to arbitrate if needed. Reasons for full-text exclusion were recorded and are presented in a PRISMA-ScR flow diagram (Figure 1).

A structured data-charting form was developed in Microsoft Excel, pilot-tested on three included sources and refined before full use. Two reviewers independently charted data from each source, with discrepancies resolved through consensus or, when required, consultation with a third reviewer. Charted variables comprised publication details and source type; country, study design and setting; caregiver sample size and demographic characteristics; caregiver–care-recipient relationship and co-residence; care recipient dementia type and severity; caregiving duration, intensity and burden measures; sarcopenia definitions and domains assessed; measurement instruments and thresholds; comparator groups; principal findings; authors’ reported limitations; and funding and conflict-of-interest declarations.

Formal critical appraisal was not undertaken because the purpose of the review was to map the extent, characteristics and methodological gaps of a small and heterogeneous evidence base rather than to estimate pooled effects, prevalence or certainty of evidence. This decision is consistent with JBI scoping review methodology, which does not mandate critical appraisal as a prerequisite for evidence mapping. Nonetheless, readers should note that the absence of formal quality assessment means that findings from methodologically weaker studies (e.g., those with small convenience samples or unvalidated outcome measures) carry equal weight in the narrative synthesis. This should be borne in mind when interpreting the reported patterns. Findings were summarised descriptively in tables and synthesised narratively according to the sarcopenia-related domains assessed, measurement approaches used and principal patterns and gaps across the included sources. Accordingly, no conclusions were drawn regarding causality or comparative effectiveness.

## 3. Results

### 3.1. Study Selection

The searches identified 946 records. After removal of 247 duplicates, 699 unique records underwent title and abstract screening, of which 685 were excluded. A total of 14 reports were sought and successfully retrieved for full-text assessment. Eight reports were excluded: three did not report a sarcopenia-related outcome and five did not examine the eligible target population. Six sources met the eligibility criteria and were included in the review (Figure 1). Four were cross-sectional studies, one was a retrospective cohort and one was a prospective cohort.

### 3.2. Characteristics of Included Studies

A summary of the included studies is provided in Table 1. Sample sizes varied widely, from *n* = 15 caregiver–care-recipient dyads to *n* = 921 older women in a U.S. cohort. Two studies compared caregivers with non-caregiving controls; the others were single-group analyses. Caregivers were predominantly female (>65%) and older, though mean ages differed by setting: a Spanish case–control study [9] enrolled female caregivers aged 50–75 years caring for a relative with moderate dementia, whereas a U.S. pilot involving caregiver–care-recipient dyads [10] reported caregiver mean age 60.4 ± 13.7 years. Two studies focused on very old spousal caregivers (mean age ≈ 80 years). All studies recruited community-dwelling caregivers providing unpaid care for relatives with dementia; one U.S. study [17] additionally included caregivers of traumatic brain injury patients but analysed dementia caregivers separately.

None of the studies applied consensus sarcopenia criteria (EWGSOP2/AWGS) or measured appendicular muscle mass. Instead, they assessed individual components such as handgrip strength, walking speed, or endurance (see Table 2). Handgrip strength was measured in four studies using dynamometers; gait speed or aerobic endurance was assessed in three studies via timed walks or the six-minute walk test. Two studies evaluated frailty using the Fried phenotype, which incorporates grip strength and gait speed cut-offs. It should be noted that the Fried phenotype, while incorporating grip strength and gait speed, is a frailty instrument rather than a sarcopenia diagnostic tool. Its use as a proxy for sarcopenia-related domains is an acknowledged limitation of the available literature, and findings derived from frailty measures should not be interpreted as equivalent to confirmed sarcopenia. No study used imaging or bioimpedance to quantify muscle mass.

### 3.3. Synthesis of Key Findings

The six studies collectively suggest that informal dementia caregivers commonly experience deficits in muscle function and mobility, although findings vary by population and outcome (Table 3). Several recurring themes emerged:

1. High prevalence of pre-frailty in older caregivers: In a Belgian cohort [12] of 82 spousal caregivers (mean age 80 years), 72% were pre-frail at baseline, and one-third progressed to worse frailty status over 16 months. Similarly, a Brazilian cross-sectional study [11] of 65 caregivers found 72.3% pre-frail and 13.8% frail using Fried’s phenotype, with frailty strongly associated with being unmarried, depressive symptoms, cognitive impairment, caring for a parent rather than a spouse, and higher caregiver burden scores. This study was excluded as there was no data pertaining to the demographics of the care recipients, and it was uncertain if care recipients had underlying dementia. Nonetheless, these data indicate that older caregivers frequently sit on the cusp of frailty, with psychosocial stressors contributing to physical decline.
2. Aerobic endurance and strength deficits: The most pronounced deficits were seen in studies using comprehensive fitness batteries. In a U.S. pilot [10] of 15 caregiver–care-recipient dyads, every caregiver scored below criterion-referenced thresholds for aerobic endurance on the six-minute walk test, and 80% of caregivers were below the threshold for upper-body strength. All female caregivers walked slower than age-adjusted norms and 60% of caregivers were below normative values for strength. Qualitative reports indicated that half of caregivers had difficulty completing physical tests, underscoring poor fitness and endurance. In contrast, an older Spanish case–control study [9] reported that female caregivers had stronger hand and leg muscles and lower waist–hip ratios than age-matched non-caregivers, although they exhibited poorer trunk extensor endurance and substantially lower mental health and general health scores. These divergent results may reflect differences in caregiver age and baseline health: mid-life caregivers may maintain or even improve limb strength through caregiving tasks, whereas very old caregivers exhibit broad deficits across endurance and strength domains.
3. Metabolic and inflammatory influences on gait: A retrospective U.S. cohort [13] of 921 women found that caregiving alone did not predict walking-speed decline over one year, but caregivers with multiple metabolic syndrome indicators experienced markedly greater declines in walking speed than those without metabolic risk. Long-term caregivers with three to four metabolic indicators declined by approximately 0.10 m/s, and dementia caregivers with high metabolic risk declined by 0.155 m/s. Another study [17] examined whether inflammation moderated the association between gait speed and cognition in 43 caregivers (mean age 58 years). Faster gait was associated with better executive function and processing speed at low or average levels of interleukin-6 and C-reactive protein, but this relationship disappeared at high inflammation levels. These findings suggest that cardiometabolic and inflammatory status modify how physical performance translates to functional outcomes in caregivers.
4. Mixed evidence on handgrip strength and nutrition: Handgrip strength results were inconsistent. In the Spanish case–control study [9], caregivers exhibited slightly higher grip and leg strength than controls, whereas in the Brazilian frailty study [11] 55% of caregivers met Fried’s criterion for weakness and 47% had slow gait. A U.S. thesis [14] analysing 47 caregivers reported a mean handgrip z-score of +0.81 (approximately 32 kg), and only two participants met sarcopenic grip cut-offs. Importantly, protein intake (mean 0.86 g/kg/day) was not correlated with handgrip strength (r = 0.089, *p* = 0.56). Nutrition status assessed by the Mini-Nutritional Assessment correlated with handgrip strength among female caregivers (r = 0.398, *p* < 0.05) but not among males. Taken together, these findings imply that while some caregivers maintain normal hand strength, older or burdened caregivers often exhibit weakness, and dietary protein alone does not account for strength variations.
5. Lack of muscle mass assessments or interventions: None of the included studies measured muscle mass or implemented exercise or nutritional interventions aimed at preventing sarcopenia. Physical performance and strength were used as proxies, leaving the prevalence of true sarcopenia unknown. Moreover, no trials evaluated the efficacy of resistance exercise or dietary supplementation for caregivers, representing a significant research gap.

These themes indicate that informal dementia caregiving is associated with high rates of pre-frailty, endurance and strength deficits, and vulnerability to metabolic and inflammatory influences. However, evidence is heterogeneous and limited by small samples and cross-sectional designs.

Across studies, sarcopenia-related risk appears to vary according to caregiver age, metabolic profile, and measurement approach rather than caregiving exposure alone. Mid-life caregivers in the Spanish case–control study demonstrated stronger limb strength than non-caregivers [9], whereas very old spousal caregivers in the Belgian cohort showed high rates of pre-frailty and progression over time [12]. In the U.S. cohort, caregiving did not independently predict gait decline, but metabolic syndrome indicators markedly amplified walking-speed deterioration among caregivers [13], highlighting the interaction between biological vulnerability and caregiving stress. The pilot fitness study and the thesis analysis further suggest that endurance and performance deficits are more pronounced than isolated handgrip weakness [10,14]. These patterns indicate that caregiver muscle health is heterogeneous and shaped by age-related physiological reserve, inflammatory load, and metabolic risk.

Future research should apply standardised sarcopenia criteria, measure muscle mass, and explore interventions to preserve or improve caregivers’ muscle health.

## 4. Discussion

This scoping review identified six sources examining sarcopenia-related domains among informal caregivers of persons living with dementia. None applied a consensus sarcopenia definition or measured appendicular muscle mass; consequently, the prevalence of sarcopenia in this population remains unknown. The available evidence suggests possible limitations in selected domains, including endurance, gait and frailty status among some older caregiver groups. However, findings for muscle strength were inconsistent, and the small, heterogeneous and predominantly observational evidence base does not establish that dementia caregiving independently causes or accelerates sarcopenia.

Several plausible pathways may link prolonged dementia caregiving with sarcopenia-related decline, although direct mechanistic evidence in this population remains limited. Chronic psychological stress, as experienced by dementia caregivers, activates the hypothalamic–pituitary–adrenal axis, leading to sustained hypercortisolism, which promotes muscle protein catabolism and inhibits anabolic signalling [7,8]. Concurrently, pro-inflammatory cytokines such as interleukin-6 and tumour necrosis factor-alpha, which are elevated in chronic stress states, lead to impaired skeletal muscle protein synthesis and accelerate proteolysis [7,17]. Mitochondrial dysfunction and increased oxidative stress, both consequences of chronic inflammation and ageing, further compromise muscle fibre integrity and regenerative capacity. Nutritional inadequacy, as suggested by the malnutrition data in one included study, may compound these effects by limiting substrate availability for muscle protein synthesis [14]. Together, these stress-endocrine, inflammatory, and nutritional pathways provide a coherent biological rationale (Figure 2) for the sarcopenia-related deficits observed in some caregiver groups, even though direct mechanistic evidence in caregivers is not yet available.

It is important to emphasise that these mechanistic pathways are inferred from established stress-endocrine and inflammation literature rather than demonstrated causally in dementia caregivers. None of the included studies applied consensus sarcopenia criteria (EWGSOP2/AWGS), none measured appendicular muscle mass, and only two assessed biological markers. As a scoping review, our aim is to map existing evidence rather than infer causality. Accordingly, mechanistic interpretations in this review are hypothesis-generating and intended to guide future research rather than to imply established pathways.

Sarcopenia is a more targetable condition than frailty. Frailty is a multi-dimensional syndrome (encompassing weakness, weight loss, slowness, exhaustion, and low activity), which indeed appears common in dementia caregivers. However, frailty can result from various organ system deficits, whereas sarcopenia zeroes in on the musculoskeletal decline that underpins physical frailty. By isolating muscle mass and strength for study, we gain objective metrics (kilograms of grip force, gait speed in m/s) and consensus cut-offs that can be tracked and improved. Moreover, sarcopenia often precedes overt frailty—it is an earlier warning sign that can be addressed before a caregiver becomes fully frail. Sarcopenia is modifiable: resistance training can increase muscle strength and mass even in late life, and nutritional supplementation (protein, vitamin D) can enhance these gains. Frailty, once established, is harder to reverse. Thus, focusing on sarcopenia aligns with a preventive, proactive approach to caregiver health. If we can identify caregivers with emerging sarcopenia (low grip strength or slowing gait), we might intervene to prevent progression to frailty or disability.

Caregivers frequently exhibit pre-frailty and fitness deficits. One cross-sectional survey [14] and one longitudinal cohort [12] reported that more than 70% of older caregivers were pre-frail and between 14% and 34% progressed to frailty over follow-up. In a small pilot of caregiver–care-recipient dyads [10], all caregivers scored below age-adjusted norms for aerobic endurance, and most performed poorly on strength tests. Even when handgrip strength remained within normal limits for many caregivers, deficits in endurance, gait and trunk muscle performance were apparent. Collectively, these findings suggest that the physical demands and chronic stress of caregiving may erode endurance and mobility before overt weakness develops. This suggests a window of opportunity for prevention if we focus on muscle function. By contrast, had we only looked at frailty as an outcome, we might conclude “some caregivers are frail,” without a clear pathway to intervene. Sarcopenia as a construct gives us that pathway (exercise, diet, lifestyle changes).

Psychosocial factors and comorbidities modulate physical decline. The Belgian [12] study linked frailty progression to depression, cognitive impairment, high caregiver burden and lack of spousal support. Metabolic syndrome indicators and systemic inflammation further modified the relationship between physical function and outcomes: caregivers with multiple metabolic risk factors experienced greater declines in walking speed, and those with elevated interleukin-6 or C-reactive protein did not show the usual positive relationship between gait speed and cognitive performance. These findings raise the possibility that caregiver sarcopenia may be intertwined with metabolic health, inflammation and mental well-being, though the cross-sectional and observational nature of the available evidence precludes causal inference.

Mixed results on handgrip strength emphasise heterogeneity. Handgrip strength—a common surrogate for muscle strength—yielded divergent results. Spanish caregivers [9] displayed stronger hands and legs than matched controls and U.S. caregivers had mostly normal grip but poor endurance [10] and no link between protein intake and strength [14]. These discrepancies likely reflect differences in caregiver age, occupation, baseline health and cultural contexts. They also caution against using grip strength alone to infer sarcopenia in caregivers without assessing endurance or muscle mass. Furthermore, the Spanish study [9] also noted that despite better muscle strength, they had worse muscular endurance. This could indicate an early stage of decline—not full-blown frailty, but specific deficits likely attributable to caregiving strain. Intervening at that stage (e.g., core strengthening exercises to improve back endurance) might prevent further decline. The substantial heterogeneity across included studies, in terms of sample size, caregiver age, geographic and cultural context, outcome measures, and comparator groups, fundamentally limits the ability to draw generalisable conclusions. For instance, findings from a small U.S. pilot of 15 caregiver–care-recipient dyads [10] cannot be meaningfully compared with those from a retrospective cohort study drawing on 921 older women, of whom 338 were caregivers [13]. Similarly, the divergent grip strength findings between Spanish mid-life caregivers [9] and older U.S. caregivers [10,14] likely reflect population-level differences rather than inconsistency in the caregiving effect per se.

Methodological limitations impede definitive conclusions. No study applied consensus sarcopenia definitions or measured appendicular muscle mass, leaving true sarcopenia prevalence unknown. The absence of a shared diagnostic framework means that ‘sarcopenia-related findings’ across studies are not directly comparable. A caregiver classified as having low grip strength in one study may not meet the threshold used in another, and neither may it reflect true sarcopenia as defined by EWGSOP2 or AWGS. This fundamentally limits the ability to estimate prevalence or pool findings. Most were cross-sectional with small samples, limiting causal inference and generalisability. Studies often lacked detailed information on caregiving intensity (hours/day), dementia severity, or physical activity levels, factors that could influence muscle outcomes. Only one study [17] examined biological markers (IL-6, CRP), and none tested interventions to improve caregiver muscle health. The heterogeneity of methods and outcomes precluded meta-analysis.

This review also has several process-level limitations. The search was restricted to publications from 2000 onwards and did not include databases such as Embase, CINAHL or Web of Science. Although no language restriction was applied at the eligibility stage, the search strategies were developed using English-language terms. Grey-literature searching was limited to ProQuest Dissertations & Theses Global and medRxiv, and relevant unpublished or non-indexed evidence may therefore have been missed. Given the scoping purpose of the review, no formal critical appraisal was undertaken; consequently, the findings map the extent and characteristics of the evidence but should not be interpreted as estimates of prevalence, causality or certainty.

Despite these issues, a consistent signal emerged: caregivers, on the whole, are a physically vulnerable group. Nearly every study identified some aspect of poorer physical health—be it endurance, strength endurance, frailty status, or nutritional status—in caregivers. Even when baseline strength was equal to controls, other metrics (back endurance, SF-36 general health) were worse. This aligns with broader literature that caregivers often neglect their own health needs, leading to chronic health impairments.

Clinicians caring for dementia dyads should consider screening caregivers for physical decline (e.g., simple gait speed or grip strength tests) and refer those at risk to exercise or rehabilitation programs. Psychosocial supports that reduce depression and burden may indirectly benefit physical health. Importantly, future research should adopt standardised sarcopenia criteria, include measures of muscle mass, and explore interventions combining resistance exercise, balance training and nutritional support tailored to caregivers’ constraints.

Sarcopenia in caregivers matters not just for caregivers’ own health, but also potentially for care recipients. A caregiver with poor muscle strength may struggle to safely transfer or ambulate their loved one, raising risk of falls or injuries for both parties. If caregivers maintain their muscle health, they might prolong their ability to care at home, delaying institutionalisation of the person with dementia. In an ageing society, supporting caregiver physical health (including muscle maintenance) could have broad public health benefits.

Based on our scoping review, we propose several priorities for future research on this topic. Firstly, there is a need for future studies to utilise consensus sarcopenia definitions (e.g., EWGSOP2) to determine prevalence of probable, confirmed, and severe sarcopenia in caregiver populations. Even simply measuring grip and gait and applying established cut-offs would allow comparisons to epidemiologic data. Developing a caregiver-specific risk profile for sarcopenia (e.g., incorporating caregiving hours, burden scores) could also be useful.

Longitudinal cohort studies of dementia caregivers with serial measurements of muscle mass, strength, and performance would be ideal where studies would start near the onset of caregiving (or even pre-caregiving) and follow through disease progression of the care recipient. This would clarify trajectories: do caregivers experience accelerated muscle decline over time relative to age norms? What are the time points of greatest decline (perhaps transitions to more intense care levels)?

Mechanistic pathways can also be further explored through the collection of biological samples (for inflammation, hormonal changes), body composition by DXA, and detailed lifestyle surveys in caregiver studies. Mixed methods allow the combination of qualitative research to identify barriers to exercise or nutrition that underlie some quantitative findings.

It remains unclear whether caring for someone with advanced dementia has a quantitatively greater impact on caregiver muscle health than caring for someone in early stages. Future research should stratify or at least record dementia severity (e.g., by FAST or CDR scales) and behavioural symptoms, to see if those correlate with caregiver sarcopenia outcomes. It may be that once patients are bedbound (very severe stage), caregivers do heavy lifting which might paradoxically strengthen some muscles but cause others to fatigue or get injured. These nuances warrant study.

## 5. Conclusions

In summary, informal caregivers of people with dementia face not only emotional and cognitive challenges but also may face physical health challenges that include loss of muscle strength, reduced endurance, and increased frailty risk. Our scoping review found that while research in this area is still sparse, there is sufficient preliminary evidence to warrant concern that chronic caregiving may be associated with accelerated musculoskeletal decline, though this remains to be confirmed by studies using standardised sarcopenia criteria. Sarcopenia—a potentially preventable and treatable condition—should be on the radar of clinicians, researchers, and policymakers involved in dementia care.

Moving forward, more robust studies are needed to accurately quantify the scope of sarcopenia in caregiver populations and to determine causal pathways. However, we do not need to wait for perfect data to act. Given what is already known about the benefits of exercise and nutrition in older adults, it is prudent to encourage and facilitate sarcopenia prevention strategies among dementia caregivers. This could mean developing tailored exercise programs that caregivers can integrate into their busy routines, providing nutritional support or meal services to ensure caregivers maintain muscle-supporting diets, and systematically monitoring caregivers’ physical function during clinical encounters.

Ultimately, supporting the physical well-being of caregivers is a win–win proposition: healthier, stronger caregivers are more likely to provide better care for longer, and they will have a better quality of life themselves. Dementia caregiving will continue to be a cornerstone of long-term care in our aging societies. By recognising sarcopenia-related decline as a plausible and modifiable concern, future studies and clinical services can better identify caregivers at risk and test practical strategies to preserve physical function without overstating conclusions that the present evidence base cannot yet establish. The old adage “you can’t pour from an empty cup” holds true—caregivers need muscle strength and vitality to meet the physical demands of caregiving, and it is our responsibility as a society to help them preserve these capacities for as long as possible.

## Figures and Tables

**Figure 1:**
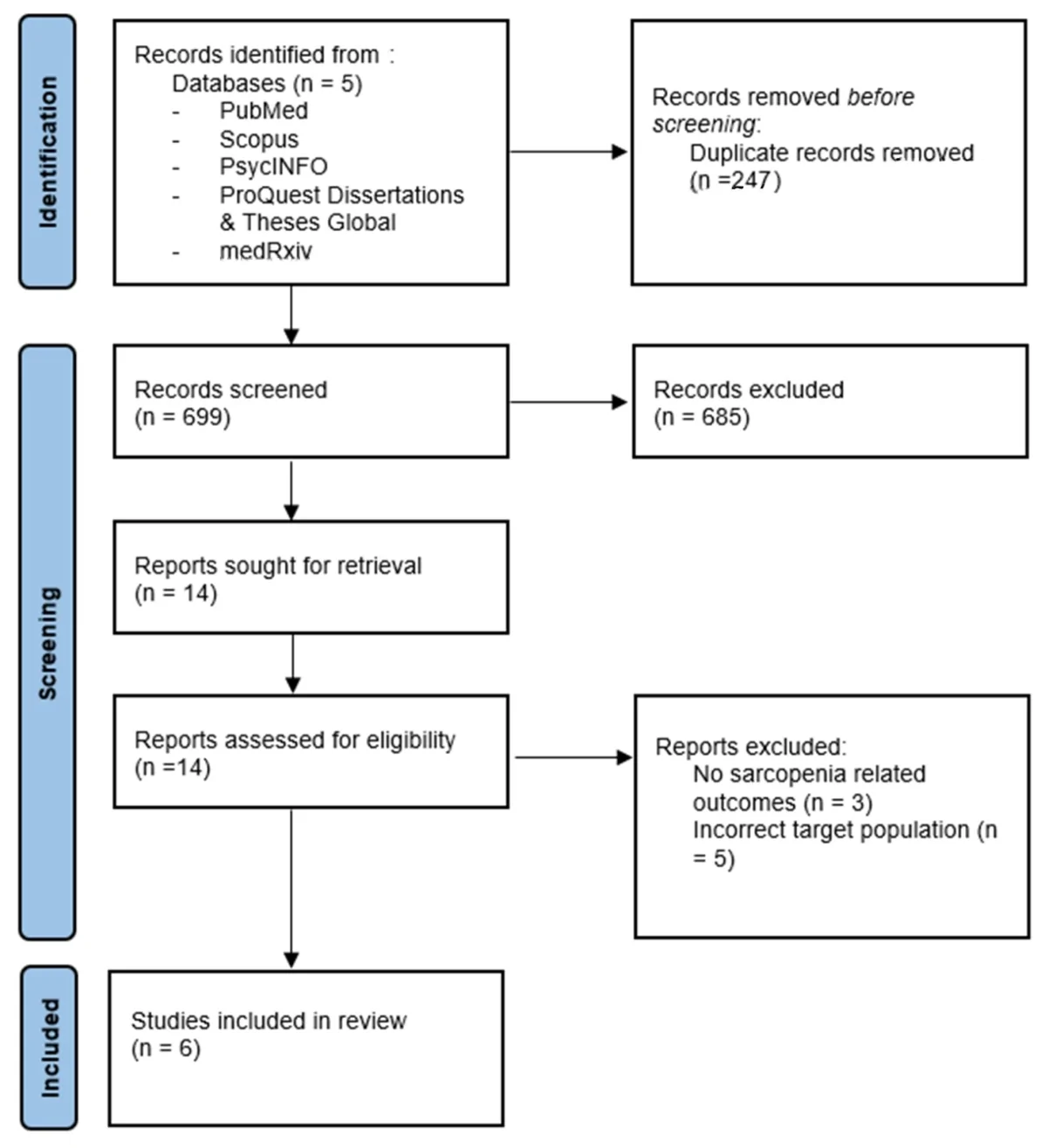
PRISMA flow diagram.

**Figure 2:**
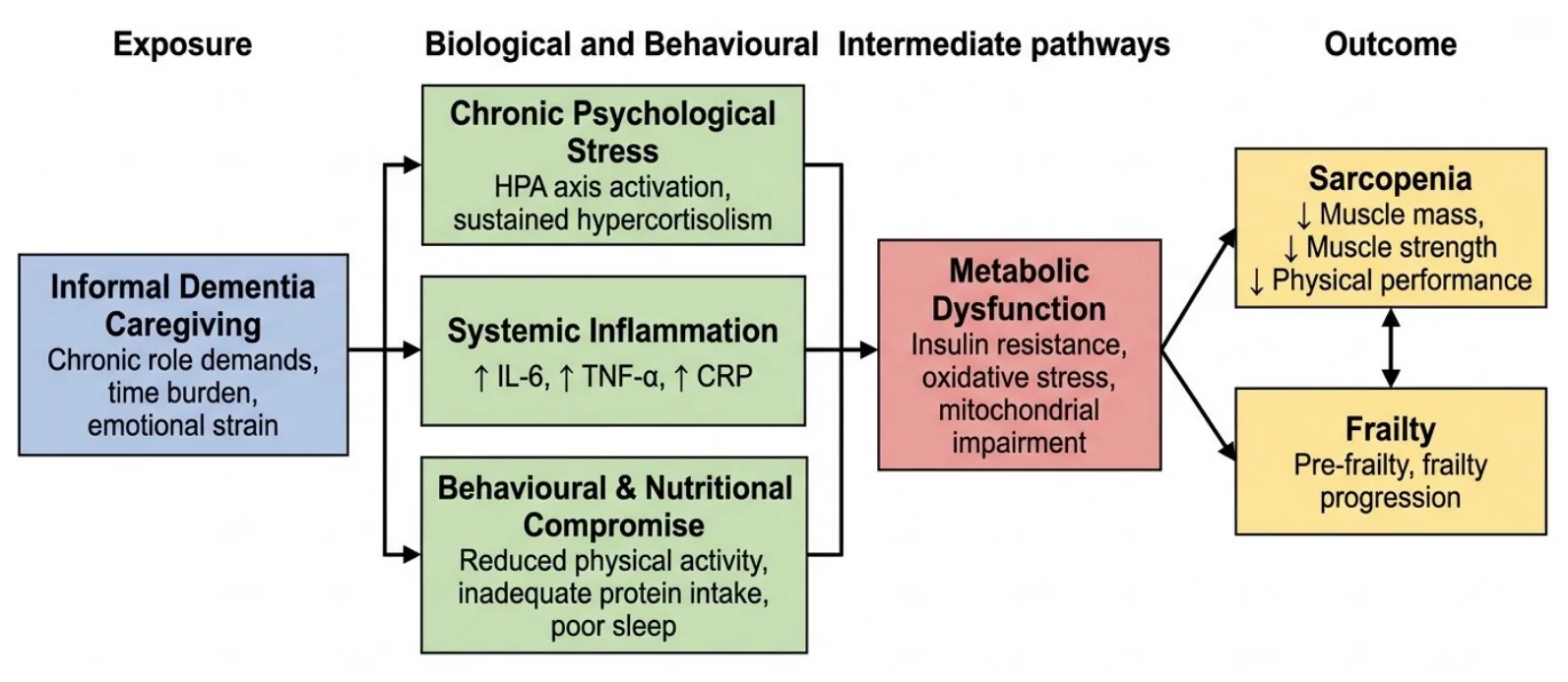
Proposed conceptual model of pathways linking informal dementia caregiving to sarcopenia risk. Arrows represent hypothesised relationships based on available evidence and biological plausibility; causal directionality has not been established in this population.

**Table 1:** Characteristics of included studies (ordered by year).

Study (Author, Year)	Country/Design	Sample (Caregivers)	Care Recipient Details	Comparison Group
Gusi et al., 2009 [9]	Spain; cross-sectional case–control	*n* = 54 female caregivers aged ≥50 y caring for relatives with dementia; recruited via regional associations	Alzheimer’s disease or other dementias, moderate stage (not quantified)	Yes—56 age-matched female non-caregivers
Fredman et al., 2010 [13]	USA; retrospective cohort (Caregiver Study of Osteoporotic Fractures)	*n* = 338 caregivers from a cohort of 921 women (mean age ≈ 81 y, 88% white); caregivers classified as long- vs. short-term; 90 cared for AD/dementia patients	Care recipients: various, including Alzheimer’s/ dementia; severity not detailed	Yes—583 non-caregiver women within same cohort
Potier et al., 2018 [12]	Belgium; prospective cohort with 16-month follow-up	*n* = 82 spousal caregivers (mean age ≈ 80 y, 54% female) of older patients with cognitive deficits or functional impairment; 78 completed follow-up	Care recipients: 83% had cognitive impairment	No external control; within-cohort frailty transitions examined
Pociask et al., 2024 [10]	USA; cross-sectional	*n* = 15 caregiver–care-recipient dyads; caregivers mean age 60.4 ± 13.7 y, 66% female	People with dementia (mean age 76.9 ± 3.9 y; mild–severe)	No control; performance compared to normative fitness standards
Rau (Thesis) 2024 [14]	USA; cross-sectional (secondary analysis)	*n* = 47 caregivers from an Alzheimer’s centre; mean age 72.9 ± 8.9 y, 57% female; predominantly white and well-educated	Persons with dementia; care recipients’ severity not reported	No control; handgrip and nutrition analysed within caregivers
Morgan et al., 2025 [17]	USA; cross-sectional secondary analysis	*n* = 43 caregivers (mean age 58.35 ± 16.3 y, 90.7% female) caring for someone with dementia or traumatic brain injury; caregivers provided ≥ 1 h/day of care and reported subjective burden	Care recipients: primarily dementia or traumatic brain injury; severity not stratified	No control; relationship between gait speed, cognition and inflammation analysed

**Table 2:** Sarcopenia-related measures used in included studies.

Study (Author, Year)	Muscle Strength Measures	Physical Performance Measures	Muscle Mass/ Biological Measures
Gusi et al., 2009 [9]	Handgrip strength (mean of both hands) using hydraulic dynamometer; leg strength via 30 s chair stand; arm strength via 30 s arm curl	Back extensor endurance test; 8-foot up-and-go for agility; chair sit-and-reach and back scratch for flexibility; no timed long-distance gait test	No muscle mass measurement
Fredman et al., 2010 [13]	Grip strength included as part of Fried frailty phenotype (cut-offs: <20 kg for women/<30 kg for men)	Walking speed over 2–3 m course measured at baseline and 1 y; decline analysed; frailty status also considered	No muscle mass measurement
Potier et al., 2018 [12]	Grip strength component of Fried phenotype; measured via dynamometer; frailty categorised as robust/pre-frail/frail	Short Physical Performance Battery (SPPB) (gait speed, chair stands, balance) and frailty transitions over 16 months	No muscle mass measurement; biological markers (IL-6, CRP, cortisol, IGF-1) assessed
Pociask et al., 2024 [10]	Grip strength measured (right and left maxima) using dynamometer; lower body strength via 30 s chair stand; upper body strength via 30 s arm curl	Six-minute walk test for aerobic endurance; 8-foot up-and-go for agility/balance; chair sit-and-reach and back scratch for flexibility	No muscle mass measurement
Rau (Thesis) 2024 [14]	Dominant handgrip strength measured; z-scores calculated; sarcopenic cut-offs applied; nutrition status correlated with grip	No gait or endurance tests; focus on grip strength only	No muscle mass measurement; dietary protein intake assessed via food frequency questionnaire
Morgan et al., 2025 [17]	No direct muscle strength tests; physical function assessed via six-minute walk (gait speed)	Six-minute walk test; gait speed used as predictor in moderation models; Stroop test measured executive function and processing speed; IL-6 and CRP assessed	No muscle mass measurement

**Table 3:** Key outcomes related to sarcopenia components in included studies.

Study (Author, Year)	Key Findings Related to Sarcopenia Components
Gusi et al., 2009 [9]	Female caregivers had lower mental health (22% lower) and general health (11% lower) than non-caregivers but displayed better body composition and stronger arm and leg muscles; however, they exhibited poorer trunk extensor endurance, suggesting specific muscle endurance deficits despite preserved strength.
Fredman et al., 2010 [13]	Caregiving by itself did not predict 1-year decline in walking speed, but caregivers with ≥3 metabolic syndrome indicators experienced greater decline. Long-term caregivers with high metabolic risk declined ~0.10 m/s and dementia caregivers with high metabolic risk declined ~0.155 m/s, indicating synergistic effects of stress and metabolic risk on mobility.
Potier et al., 2018 [12]	Among spousal caregivers (mean age 80 y), 72% were pre-frail at baseline; at 16-mo follow-up, one-third transitioned to a worse frailty state. Nutrition, cognition, Short Physical Performance Battery scores and mood were stable in ~70% of caregivers, suggesting progressive frailty occurs in a subset despite overall stability.
Pociask et al., 2024 [10]	All caregivers scored below criterion-referenced thresholds for aerobic endurance, and 80% performed below thresholds for upper-body strength. Moreover, 100% of female caregivers walked slower than age-adjusted norms, and roughly 60% were below normative values for strength. Poorer walking distance was associated with greater assistance needed for self-care ADLs.
Rau (Thesis) 2024 [14]	The mean handgrip z-score was +0.81; only two caregivers met sarcopenic grip thresholds. There was no association between total protein intake and handgrip strength (r = 0.089, *p* = 0.56). Nutrition status (Mini-Nutritional Assessment) correlated with grip strength in female caregivers (r = 0.398, *p* < 0.05).
Morgan et al., 2025 [17]	Inflammation moderated the relationship between gait and cognition. Faster gait speed predicted better executive function and processing speed in caregivers with low or average IL-6 and CRP, but the relationship vanished in those with high inflammation. This suggests systemic inflammation may suppress the cognitive benefits of mobility.

## Data Availability

No new data were created or analyzed in this study. Data sharing is not applicable to this article.

## Supplementary Materials

The following supporting information can be downloaded at https://www.mdpi.com/article/10.3390/jcm15197345/s1, Supplementary Material S1: Full PubMed search strategy; Supplementary File S1: Completed PRISMA-ScR checklist [16].
