# Supplementary material for: Sarcopenia in Informal Caregivers of Persons Living with Dementia: A Scoping Review
Source: J Clin Med. 2026 Sep 22;15(19):7345. doi: 10.3390/jcm15197345 (PMC13666299; doi:10.3390/jcm15197345)
Supplement: Supplementary file 1 [file jcm-15-07345-s001.zip › jcm-4485100-supplementary material S1.pdf]

Supplementary Material S1. Full PubMed search strategy

The search strategy was developed for PubMed using Medical Subject Headings and free-text terms relating to informal caregivers, dementia, and sarcopenia or its component domains. The strategy was adapted to the syntax and indexing of the other information sources.

Database: PubMed
Date searched: 28 June 2025
Limits: Humans; English language; publication date from 1 January 2000 to 28 June 2025

```
(
  "Caregivers"[Mesh]
  OR caregiver*[tiab]
  OR carer*[tiab]
  OR "family caregiver*"[tiab]
  OR "spousal caregiver*"[tiab]
  OR "informal caregiver*"[tiab]
  OR "unpaid caregiver*"[tiab]
)
AND
(
  "Dementia"[Mesh]
  OR "Alzheimer Disease"[Mesh]
  OR dementia*[tiab]
  OR Alzheimer*[tiab]
  OR "vascular dementia"[tiab]
  OR "frontotemporal dementia"[tiab]
  OR "Lewy body dementia"[tiab]
  OR "Parkinson* dementia"[tiab]
  OR "cognitive impairment"[tiab]
)
AND
(
  "Sarcopenia"[Mesh]
```

```
  OR sarcopenia[tiab]
  OR dynapenia*[tiab]
  OR "Frailty"[Mesh]
  OR frailty[tiab]
  OR "Muscle Strength"[Mesh]
  OR "Muscle Weakness"[Mesh]
  OR muscle mass[tiab]
  OR "lean mass"[tiab]
  OR "appendicular skeletal muscle mass"[tiab]
  OR ASM[tiab]
  OR handgrip[tiab]
  OR "grip strength"[tiab]
  OR "gait speed"[tiab]
  OR "short physical performance battery"[tiab]
  OR SPPB[tiab]
  OR "muscle cross-sectional area"[tiab]
)
AND humans[Mesh]
AND English[lang]
AND ("2000/01/01"[PDAT] : "2025/06/28"[PDAT])
```
